# Common pitfalls in drug target Mendelian randomization and how to avoid them

**DOI:** 10.1186/s12916-024-03700-9

**Published:** 2024-10-15

**Authors:** Dipender Gill, Marie-Joe Dib, Héléne T. Cronjé, Ville Karhunen, Benjamin Woolf, Eloi Gagnon, Iyas Daghlas, Michael Nyberg, Donald Drakeman, Stephen Burgess

**Affiliations:** 1 Sequoia Genetics, London, UK; 2 Department of Epidemiology and Biostatistics, School of Public Health, Imperial College London, 90 Wood Lane, London, W12 0BZ UK; 3 Cardiovascular Division, Perelman School of Medicine, University of Pennsylvania, Philadelphia, USA; 4 Medical Research Council Biostatistics Unit, University of Cambridge, Cambridge, UK; 5 School of Psychological Science, University of Bristol, Bristol, UK; 6 Medical Research Council Integrative Epidemiology Unit, University of Bristol, Bristol, UK; 7 Centre de recherche de l’Institut universitaire de cardiologie et de pneumologie de Québec, Laval University, Québec, Canada; 8 Department of Neurology, University of California San Francisco, San Francisco, CA USA; 9 Cardiovascular Biology, Global Drug Discovery, Novo Nordisk A/S, Maaloev, Denmark; 10 University of Cambridge Centre for Health Leadership & Enterprise, Judge Business School, Trumpington Street, Cambridge, UK; 11 Advent Venture Partners, London, UK; 12 Cardiovascular Epidemiology Unit, Department of Public Health and Primary Care, University of Cambridge, Cambridge, UK

**Keywords:** Mendelian randomization, Drug target, Pharmacology, Drug development

## Abstract

**Background:**

Drug target Mendelian randomization describes the use of genetic variants as instrumental variables for studying the effects of pharmacological agents. The paradigm can be used to inform on all aspects of drug development and has become increasingly popular over the last decade, particularly given the time- and cost-efficiency with which it can be performed even before commencing clinical studies.

**Main body:**

In this review, we describe the recent emergence of drug target Mendelian randomization, its common pitfalls, how best to address them, as well as potential future directions. Throughout, we offer advice based on our experiences on how to approach these types of studies, which we hope will be useful for both practitioners and those translating the findings from such work.

**Conclusions:**

Drug target Mendelian randomization is nuanced and requires a combination of biological, statistical, genetic, epidemiological, clinical, and pharmaceutical expertise to be utilized to its full potential. Unfortunately, these skillsets are relatively infrequently combined in any given study.

## Background

Drug development has a notoriously high failure rate, with less than 10% of drugs entering clinical study eventually being approved for use in patients [1]. This low success rate may be at least partly attributed to most of the preclinical evidence for drug effects coming from animal studies and traditional epidemiological associations, which are respectively limited in their translatability to humans and their ability to draw causal inferences [2].

**Fig. 1:**
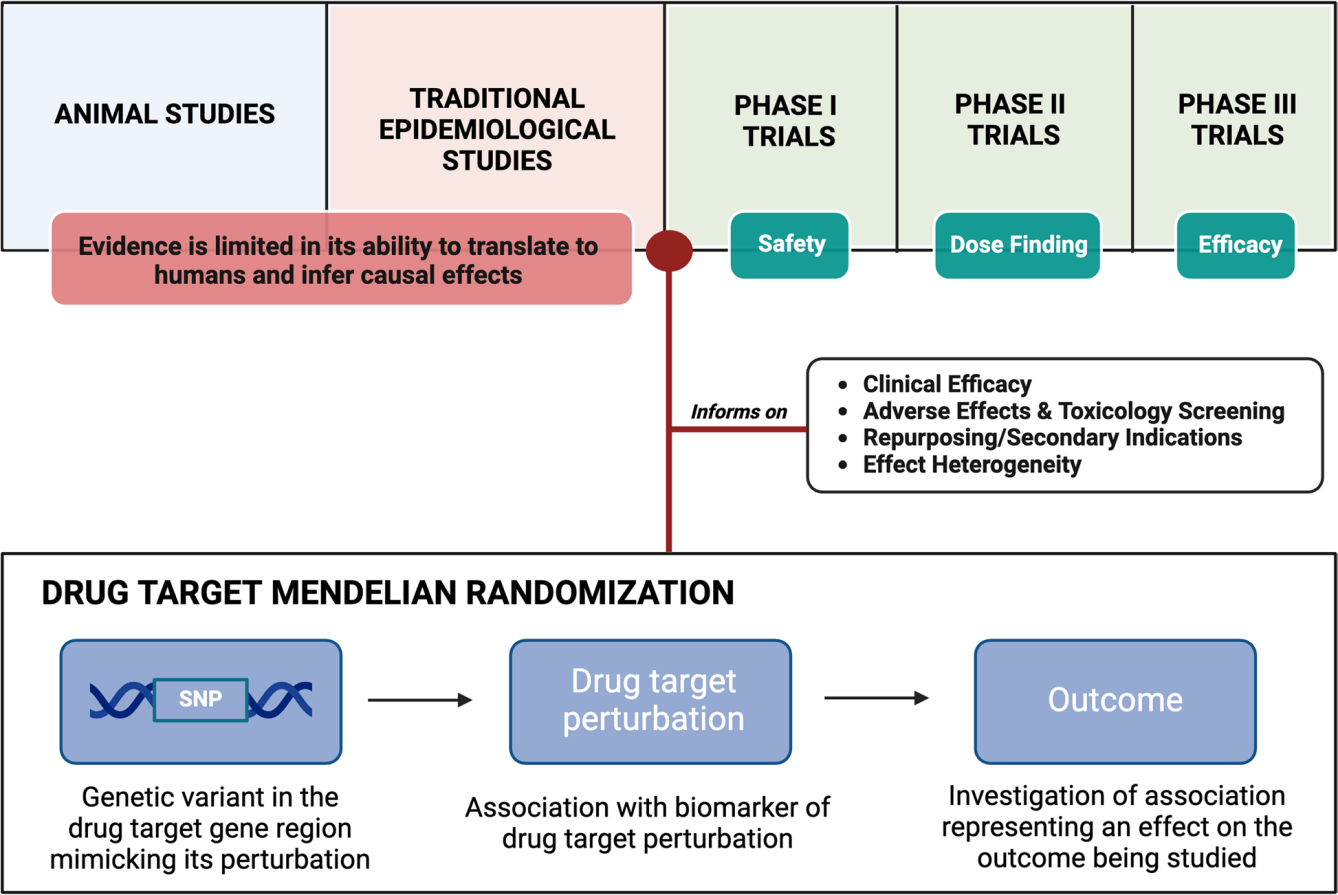
Drug target Mendelian randomization within the context of drug development

Mendelian randomization is a method that uses genetic variants as instrumental variables for studying the causal effect of an exposure on an outcome [3]. These variants should relate to the exposure of interest, and to the outcome under investigation only through the exposure, and not through alternative pleiotropic pathways [3, 4]. The Mendelian randomization paradigm helps overcome the limitations of traditional epidemiology because it uses human genetic data to infer the causal effects of drug target perturbation in humans [5, 6]. Given that most drug targets are proteins, and that proteins are coded for by genes [3], investigation of drug target effects is particularly amenable to study through genetics-based causal inference methods such as Mendelian randomization [6]. In fact, this approach is increasingly being used to study the potential effect of pharmacological perturbation of drug targets on clinical outcomes for the purposes of informing drug development strategies, a practice known as ‘drug target Mendelian randomization’ (Fig. 1) [1, 6]. Interestingly, drug targets with human genetic evidence have been shown to be at least twice as likely to make it through clinical development [7, 8]. Given that the average new drug requires more than 10 years and 1 billion US dollars to obtain regulatory approval [9], insights from Mendelian randomization studies have the potential to tremendously improve the efficiency of generating effective treatments to prevent and treat disease.

Judicious use of human genetic data has the potential to inform on various critical aspects of drug development, including on-target efficacy, safety, repurposing, biomarker selection, effect heterogeneity, and interactions [10]. Given the potential impact on informing drug development efforts, and in conjunction with increasing data availability, it is not surprising that there has been an explosion in the number of drug target Mendelian randomization analyses performed over recent years, and that most large pharmaceutical companies now incorporate human genetic evidence in their drug development pipelines [11]. However, the increasing number of drug target Mendelian randomization analyses has, in some cases, not been accompanied by a corresponding improvement in quality, resulting in increasing uncertainty around their utility and translatability.

The purpose of this review is to share insights gained from over a decade of experience in drug target Mendelian randomization, through which we have actively contributed by publishing numerous methods-focused and applied research papers. The intended audience for this review includes experts in the field of Mendelian randomization who are interested in effectively applying methods for maximal clinical impact, and individuals in the drug development field who are in the pursuit of leveraging genetics-based insights. Given the relatively recent emergence of this paradigm, we are all on a steep learning curve, and it is therefore important for us to share our knowledge along the way. This review is not meant to be a prescriptive set of guidelines [12, 13], a repetition of the general principles of Mendelian randomization [14, 15] and drug target Mendelian randomization [1, 6, 16], or a statistical handbook on performing these analyses [17, 18], as these topics have already been extensively covered previously. The aim of this review is rather to share our learnings effectively, describing what we have found to be the common pitfalls in drug target Mendelian randomization, along with strategies to help avoid them. We acknowledge that we have previously published studies that directly contradict some of our current recommendations [19, 20]. However, we consider this to be a hallmark of science—a process that involves the intricate refining ideas and concepts through a continuous cycle of learning and improvement. We hope to utilize our insights in this review to advance the applications of drug target Mendelian randomization within the scientific community.

We divide the aforementioned pitfalls into sections related to (i) selecting exposures specific to the research question, (ii) identifying biologically plausible instruments for the exposure under study, (iii) utilizing appropriate outcome genetic association data, and (iv) defending against possible false negative and false positive results. We then offer some advice on general considerations when contextualizing a drug target Mendelian randomization study, before providing our conclusions on the current state of the field, its gaps, and future directions.

### Selecting exposures specific to the research question

Misspecification of the exposure relevant to the research question is perhaps the most fundamental cause of a drug target Mendelian randomization analysis giving misleading results. A requisite for success in defining the exposure is an understanding of the mechanism of action for the drug target under investigation. This can then enable identification of appropriate biomarkers for the exposure, to thus help identify suitable instruments.

#### Drug targets with multiple mechanisms

Drugs exerting their effects via multiple mechanisms are not necessarily amenable to drug target Mendelian randomization, or require thorough considerations of all the pathways they involve. To illustrate this, one Mendelian randomization study claimed to be investigating the effect of metformin, a commonly used anti-diabetic drug [21]. However, the actual analysis performed was concerned with the effects of genetically predicted growth differentiation factor 15 levels, whose altered circulating levels represent only one possible mechanism by which metformin exerts its effects [22, 23]. Consideration of the various targets through which metformin exerts its effects may have yielded contrasting results that are more reflective of its various mechanisms. Furthermore, in instances where the drug target is not known, as is the case for even some drugs routinely used in clinical practice [24], drug target Mendelian randomization will not be viable.

#### Drug use versus drug target effects as exposures

Another example of this is consideration of drug use (e.g. electronic health care drug prescription records or self-reported medication use), rather than the mechanisms through which the drug target elicits its clinical effects (e.g. reduction in systolic blood pressure through calcium channel blockade), as the exposure of interest. Genetic predictors of drug use are more likely to relate to the underlying condition that the drug is used to treat, socioeconomic factors related to medication access, or to generic determinants of medication adherence, rather than the effects of the drug target [25, 26]. In contrast, genetic variants predicting drug target effects would be expected to specifically mimic pharmacological perturbation of the protein target, rather than factors that reflect its use. This discrepancy has been studied in detail for caffeine, where genetic variants predicting the effects of plasma caffeine levels yield different Mendelian randomization estimates compared to those predicting its consumption [27]. These examples reinforce the importance of asking the right research question, and carefully selecting relevant exposure data.

#### Multi-protein complex drug targets

Situations where the drug target is made up of multiple proteins can become particularly challenging. For example, antihypertensives of the calcium channel blocker class target several protein subunits that collectively form the calcium channel. Mendelian randomization analyses of this drug target have pooled variants in the genes coding for any of the protein subunits to generate instruments for drug target perturbation [28–30]. However, this does not account for unequal contributions of the different protein subunits to the target’s overall effects, nor potential interactions between them.

#### Non-protein drug targets

There is even greater complexity when the therapeutic target is not a protein. For example, some Mendelian randomization investigations into the effect of increasing blood metabolite levels have considered genetic variants from the entire genome as instruments [31]. These will likely represent heterogeneous mechanisms, with potentially distinct effects. Interpreting which of these mechanisms or pathways are most representative of the therapeutic intervention being considered will require biological insight into how the different genetic variants may be exerting their effects, in relation to the intervention under study.

#### Drug targets with no valid genetic instruments

Not all drug targets, nor therapeutic interventions, may be amenable to study using Mendelian randomization. In our experience, genetic instruments are identifiable for the majority, but not all drug targets. This is broadly consistent with the estimated proportion of two thirds of approved drugs that have genetic support [32]. This is likely partially attributable to the lack of available genetic proxies for the remaining third of drug targets. Examples of drug classes that have recently been approved but have not been robustly instrumented in Mendelian randomization are sodium-glucose co-transporter 2 inhibitors and interleukin-17 inhibitors. Drug target Mendelian randomization can only be used to study host protein drug targets, and so is not applicable to the study of pharmacological agents directly targeting microorganisms. In this way, drug target Mendelian randomization successfully predicted immunomodulatory drug targets that would be efficacious in severe covid-19 [33], but not successful antiviral agents.

#### Long versus short-acting pharmacological perturbation

Genetic variants typically predict lifelong changes in drug targets, and so tend to resemble the effect of long-term pharmacological perturbations. This is an important consideration in scenarios where the effects of long-term pharmacological perturbation are different to those with short-acting mechanisms. An example of this is glucose-dependent insulinotropic polypeptide receptor (GIPR) signalling, where in some tissues the effects of long-term, in contrast to short-term, agonism are believed to mimic antagonistic effects via receptor desensitization [34].

### Biological considerations when selecting instrument variants

Our approach for undertaking a drug target Mendelian randomization study almost always begins with a detailed interrogation of the known biology of the target being investigated. In this way, it is possible to optimize instrument selection by exploring the possible data sources that may be relevant to use. We recommend some considerations in this section.

#### Acknowledging the limitations of expression and protein quantitative trait loci

Instruments in Mendelian randomization analyses should associate with the exposure and only associate with the outcome through the exposure and not some alternative pleiotropic pathway [35, 36]. In drug target Mendelian randomization, the exposure is the perturbation of a drug target. There are few genetic association data sets available that directly measure drug target perturbation, so it is necessary to use surrogates or ‘proxies’ for this. It has become common practice to use genetic variants associated with gene expression or protein abundance as such proxies. These are called expression quantitative trait loci (eQTLs) for gene expression and protein quantitative trait loci (pQTLs) for protein expression. The advantages of using these traits to select instruments are that such data are widely available [37–41], and this approach requires little biological insight to be blindly applied at scale [42–44]. Superficially, this strategy also makes sense, because variants associating with altered levels of the drug target would be expected to serve as instruments for its perturbation. Unfortunately, the reality is quite different for several critical reasons.

Firstly, genetic variants predicting gene expression typically relate to transcription of a gene region and affect expression levels of several genes simultaneously [45], thereby introducing inherent pleiotropy that risks biasing Mendelian randomization analyses. This issue was likely why a drug target Mendelian randomization analysis of fibroblast growth factor 21 gave misleading results [46], with unintentional flipping of the direction of association [47]. Secondly, such genetic predictors of gene and protein expression can be tissue specific [48]. Indeed, this heterogeneity in the genetic predictors of gene and protein expression across different tissues has allowed for multivariable drug target Mendelian randomization analyses that investigate the specific tissue in which a target is likely exerting its clinical effects [49]. However, it also means that using genetic association estimates from mis-specified tissues will yield potentially misleading results [50]. This is challenging considering that tissues driving disease associations can be unknown, especially when dealing with circulating factors. Thirdly, genetic predictors of gene and protein expression may vary in different physiological states [51], and data related to the specific context under investigation are scarcely available. Fourthly, there are issues around the specificity and sensitivity of assays used to measure gene expression and relative protein abundance which can limit the validity of any consequently identified genetic associations [52].

For these reasons, we recommend some degree of biological validation of instruments when they are selected using gene expression or proteomic data. A good example is the study of angiotensin converting enzyme (ACE) inhibition, whereby instruments were selected and validated by their associations with lower *ACE* gene expression and plasma ACE protein levels, as well as with lower blood pressure, a known clinical effect of ACE inhibition [53]. Similarly, in a study of phosphodiesterase type 5 inhibition, instruments were selected and validated using their association with lower gene expression, as well as lower blood pressure, and lower risk of erectile dysfunction and pulmonary hypertension, consistent with the known clinical effects of perturbing this drug target [54].

The use of clinical traits, biomarkers, and endophenotypes related to established or biologically expected drug target effects for identifying instruments offers other critical advantages over the use of gene expression or proteomic data. Specifically, there is greater potential for the link between a variant in the protein coding gene and the mechanism of action relevant to the clinical outcome being lost or confounded by pleiotropic associations when the biological distance from the variant to the trait used to select instruments is shorter (Table 1). This risk is reduced by identifying instrument variants using clinically relevant traits that are further along the putative causal pathway, and hence more proximal to the outcome under investigation. As such, clinical traits related to the drug target should generally be preferred for selecting instrument variants over molecular traits. A challenge to this approach is that it requires biological understanding of the drug target and is therefore more difficult to scale in agnostic hypothesis-free analyses.

**Table 1:**
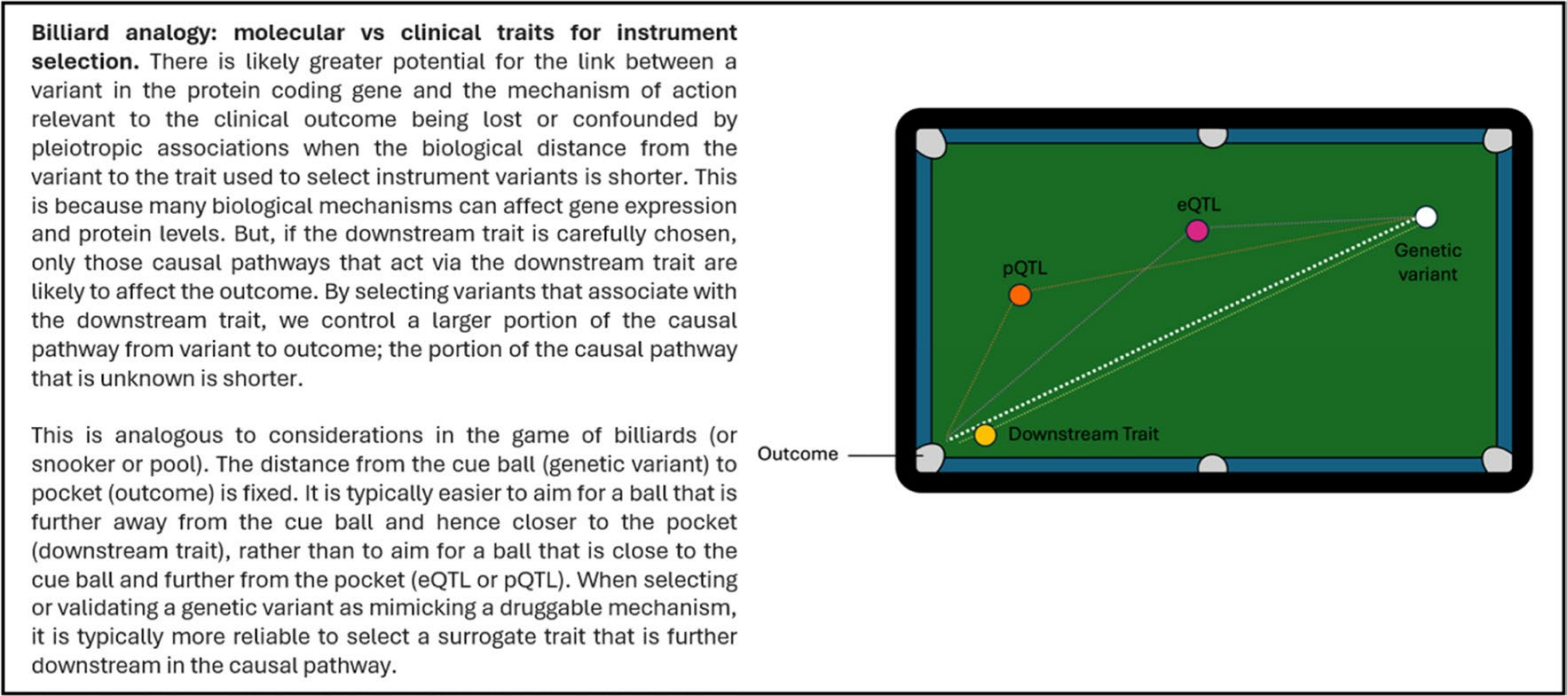
Billiard analogy

#### Considerations for protein function altering genetic variants

Other strategies for selecting instruments in drug target Mendelian randomization include variants that have effects on gene or protein function, rather than levels. Such instruments may be described as ‘loss-of-function’ or ‘gain-of-function’ variants. In practice, such terminology is potentially misleading, as protein function is scarcely describable on a binary or linear scale and is more commonly multi-dimensional. For example, for a protein that serves both as a cell surface receptor and circulating soluble receptor (e.g. interleukin-6 receptor), its reduced binding to the cell surface may increase some of its activities while reducing others. An example of a genetic instrument that exerts functional effects is a missense variant in the *GIPR* gene, which has been used to mimic long-term functional antagonism [55–57], although its biological effects are somewhat more complicated than this [34]. As with eQTLs and pQTLs, we similarly advocate validation of instrument selection using functionally annotated variants with biologically informed positive control associations. For the GIPR example above, the described missense variant used as an instrument for inhibition also strongly associates with lower body weight, consistent with what has been demonstrated in clinical trials [58]. Overall, it is important that as much evidence is obtained as possible to support the validity of the instruments employed, as without valid instruments, the whole Mendelian randomization study will be misleading.

#### Incorporation of biological knowledge

In conclusion for this section, incorporating biological insights can be transformative for selecting appropriate and valid instruments. Even when instruments are derived from eQTL or pQTL data, pre-acquired biological insight would enable the selection of the appropriate tissue to consider [38]. Being aware of all options also allows for the most relevant data with greatest statistical power to be leveraged, along with as many traits as possible, to maximize interrogation of instrument validity. For example, drug target Mendelian randomization analyses of interleukin-6 receptor signalling leveraged genetic variants robustly associated with several biomarkers known from clinical practice to be affected by perturbation of this drug target [59]. While this instrument has also been validated through positive control associations with disease outcomes known to be affected by inhibition of this target [60], such a rigorous approach may not be feasible with novel drug targets.

Having identified genetic instruments to study pharmacological perturbation of the drug target under consideration, the next step is a thorough understanding of the outcome of interest. This will consequently allow for the relevant outcome genetic association data to be selected or compiled, along with the most appropriate statistical analysis plan. Every drug target is unique, and every research question is different, so such considerations cannot be easily automated to cater for all scenarios.

### Outcome definition

As with all Mendelian randomization studies, it is important for the outcome under study to be suitably defined. In some scenarios, there may not be appropriate genetic association data available for the desired outcome, or at the desired level of phenotypic granularity. Therefore, it may be necessary to resort to the next best data source available. For example, heart failure is a heterogeneous disease with many distinct aetiological mechanisms, which vary in their treatment [61]. However, the most utilized genetic association data only consider heart failure as one entity [62]. The situation is similar for studies of chronic kidney disease [63]. Other common challenges in outcome definitions relate to misclassification, both in self-reported and clinician-ascribed diagnoses [64]. Similarly, studies of outcomes that are more common in older individuals may be vulnerable to survival bias [65]. Overall, high-quality curated data is essential for the success of drug target Mendelian randomization studies.

A fundamental distinction in outcome definitions separates risk of disease incidence and disease progression. For example, glucagon-like peptide 1 agonists are licensed for the treatment of type 2 diabetes mellitus; however, genetic association data for type 2 diabetes mellitus risk specifically relate to the risk of developing the disease [66]. In this instance, the mechanisms predisposing to type 2 diabetes overlap with those affecting its progression, so the distinction is not so critical, and genetic association data for type 2 diabetes risk generally correlate well with those for glycaemic control [67]. However, in scenarios where the genetic predictors of disease risk are quite different to those of disease progression, the implications can be more pronounced. For example, ischemic stroke typically arises from an obstruction of blood flow to the brain, most commonly due to a blood clot. In contrast, progression of disability after stroke is more related to neurological and inflammatory mechanisms affecting brain injury and recovery, as well as social factors related to engagement with rehabilitation. As such, genetic association data for stroke risk cannot be used to study drug targets for stroke recovery, and vice versa. Consistent with this, there is little correlation between the genetic predictors of ischemic stroke risk and recovery [68].

### Statistical power considerations and interpretation of associations

#### Statistical power considerations

It is not uncommon for statistical power calculations to be expected when planning a drug target Mendelian randomization study. However, this would require an estimate of the proportion of variance of the drug target effect that is explained by the genetic instrument [12]. With perhaps the exception of homozygote ‘complete-loss-of-function’ mutation carriers, we are not aware of any consistent means of reliably estimating the proportion of variance in drug target effects explained by genetic variants. As such, it seems implausible to perform reliable statistical power calculations for most drug target Mendelian randomization scenarios. Further, even if such calculations could be performed, it is not clear how they would translate clinically, as the effect of any drug compound or therapeutic intervention in clinical practice would be a function of the pharmacological properties of that compound, including both its pharmacodynamic and pharmacokinetic characteristics.

Instead, to gauge the relative power of drug target Mendelian randomization analyses, it may be more appropriate to compare estimates against those obtained from similar analyses of related traits. For example, in a drug target Mendelian randomization study into the potential repurposing opportunity and adverse effects of commonly prescribed antihypertensive drug classes, comparisons were made to the Mendelian randomization analyses of genetically predicted lower blood pressure by any mechanism after sampling the same number of variants [69]. An analogous approach was taken in a study comparing effects of the two C-type natriuretic peptide receptors [70].

When interpreting the results of a drug target Mendelian randomization analysis, as with all analyses, it is important to consider that the absence of any strong associations may also be attributed to low statistical power. This may yield false negative findings. For example, there is strong genetic evidence implicating both lipoprotein lipase (LPL) and angiopoietin-like protein 4 (ANGPTL4) as therapeutic targets for lipid-lowering and cardiovascular disease reduction [71]. However, such evidence implicating angiopoietin-like protein 3 (ANGPTL3) as a target for cardiovascular disease is more limited when considering common genetic variants [72]. Given that this evidence is stronger when considering rarer, functionally relevant variants in *ANGPTL3* that have a greater magnitude of effect, we speculate that this discrepancy may be attributable to low statistical power in some of the studies [73]. Consistent with this, *ANGPTL3* is expressed in the liver and then circulates through the plasma to its relevant sites of action, while *LPL* and *ANGPTL4* are expressed more widely [74]. Thus, the ‘biological distance’ between ANGPTL3 and cardiovascular disease may be greater than for LPL and ANGPTL4, suggesting that statistical power may also be lower, even if the relative efficacy of these drug targets might be similar in clinical practice. This claim holds even if we do not measure *ANGPTL3* expression in the liver, as what is important is the distance between the mechanism of interest and the outcome, regardless of which biomarker is used to measure the mechanism.

#### Making sense of false positive findings

False positive findings are also rife in drug target Mendelian randomization. With the explosion in data availability and the increasing ease by which they may be conducted [75], an unprecedented number of analyses are being performed daily. The pressure to publish for individuals working in academia means that scientists will be eager to share any potentially noteworthy associations that they identify, including at the cost of bypassing appropriate levels of scrutiny, replication, or validation. Such studies may be ‘false positives’ because of publication bias, as well as selective sharing of significant findings without appropriate transparency or correction for multiple testing.

Other common causes for false positive findings in drug target Mendelian randomization relate to genetic confounding through variants in linkage disequilibrium. Such possibilities can be explored using statistical colocalization methods as follow-up analyses for drug target Mendelian randomization, as previously described [76]. For example, a previous study used colocalization to provide evidence that circulating GIP levels and cardiovascular disease risk are driven by distinct causal variants in the *GIPR* gene, and so any identified Mendelian randomization associations supporting an effect of GIP levels on cardiovascular disease risk at this locus are likely attributable to genetic confounding through this correlated variant [77].

Even for the most comprehensive drug target Mendelian randomization analysis, it is still critical to triangulate the evidence with all other available forms of evidence when drawing conclusions to inform drug development efforts. For example, drug target Mendelian randomization analyses have consistently generated evidence to support that cholesteryl ester transfer protein inhibition will be an efficacious strategy for reducing cardiovascular disease risk [78], yet until recently, clinical trial efforts in this area have been unsuccessful [79]. This illustrates that even if there is genetic evidence that a target is likely to be efficacious, generating a drug that achieves this result is still dependent on its pharmacological properties, the population that is treated, the timing of treatment, and the specific outcome that is studied.

### Human genetic insights in the context of preclinical evidence

In conjunction with genetic evidence, a series of preclinical validation studies are needed to provide mechanistic insight into how a novel target is linked to disease. These studies are crucial for improving confidence in a target playing a causal role but are also key for highlighting how the target should be pharmaceutically modulated. As an example, a protein may have several functions that relate to different structural elements and thus it may be pivotal to understand which epitope needs to be modulated to achieve the desired alteration of protein function.

Preclinical experiments should preferably be performed in assays comprised of human cells displaying the relevant disease phenotype. Recapitulating such phenotypes has typically been difficult. However, with the emergence of single-cell transcriptomic and proteomic profiling that includes sufficient spatial resolution, it is possible to map out the molecular fingerprint of spatially distributed cellular populations in disease tissue [80]. Here, it is important to underscore that recapitulating such a phenotype in vitro is no simple feat. This is exemplified in the case for stem cell-derived cardiomyocytes, where it has proven difficult to induce a phenotype that fully resembles that of an adult myocyte [81]. When such challenges are overcome, it is possible to explore the effects of perturbing a molecular target and thereby provide crucial insights into how this may hold a therapeutic potential in a given cell type and tissue relevant for human disease.

There may also be targets that cannot be validated in single- or even multicellular systems as the mechanism in scope can only be captured in a model system allowing for integrative responses between numerous tissues and organs. To avoid that a potential human relevant therapeutic effect is lost in translation, animal models can be used if the mechanism identified is also operating in the selected model that recapitulates key features of human disease. Such model understanding requires intricate characterization of the model and thus comes with a substantial investment in time and resources, which to some extent may explain why inadequate models are often chosen. Failure to choose an animal model system that sufficiently mimics relevant human pathophysiology and the molecular mechanism in scope is commonly seen and is a key cause of low human translatability of findings as evidenced by efforts made within the area of atherosclerosis research [82].

It is important to highlight that the information obtained from the above-described preclinical studies should be contextualized with other layers of human data. Hence, findings from preclinical experiments should be used to define and refine genetic analyses and vice versa. This iterative process will enhance the probability of success of the drug discovery program by providing the basis for asking the relevant research questions.

### Future directions

With the increasing popularity of drug target Mendelian randomization, there is also paralleled potential to optimize the quality of studies performed and the resultant insights that can be gained from them. Paradoxically, the growth in the number of published analyses has apparently not yet translated to directly informing drug target discovery and development efforts [83]. However, we acknowledge that the actual impact of genetics in this regard may be masked by a need for pharmaceutical and biotechnology companies to protect commercially sensitive findings.

Although the approach is potentially impactful, it should always be interpreted within the context of its limitations. Mendelian randomization analyses typically consider the lifelong effects of small changes arising from genetic variation, which is rarely the same thing as a discrete clinical intervention observed at a specific timepoint in the life course [6]. This is on top of the other potential limitations encountered with the method, including absence of reliable instruments, bias from genetic confounding through variants in linkage disequilibrium, and unavailability of relevant genetic association data. We emphasize that drug target Mendelian randomization should by no means be considered a substitute for comprehensive preclinical safety and toxicology assessment, or for thorough evaluation through clinical trials. Instead, it should be used in conjunction with other sources of evidence to help prioritize and inform clinical development efforts.

Historically, many drug target Mendelian randomization efforts have focused on cardiometabolic and anti-inflammatory targets. This is likely attributable to the amenability of these targets to such analyses, as well as their relevance to clinical translation efforts. In contrast, this has left gaps in other therapeutic areas, particularly related to psychiatric and cancer outcomes. For psychiatric outcomes, this may be explained by the complexity of the central nervous system and related challenges in identifying valid instruments and designing translatable drug target Mendelian randomization analyses. For cancer outcomes, most malignancies arise due to somatic mutations, whereas Mendelian randomization is typically concerned with the effects of germline genetic variation. Additionally, most genetic association data for cancer and psychiatric outcomes relate to disease incidence rather than progression, whereas most of their therapeutics are focused on disease treatment rather than prevention. For these reasons, further efforts are required to understand the degree to which pharmacological targets for prevention are also applicable to disease treatment, and vice versa.

With the continued access to larger, more diverse, and higher-quality genetic association and epidemiological data, in conjunction with advancing statistical methods and the emergence of artificial intelligence, there has never been a more exciting time for the field of drug target Mendelian randomization. While artificial intelligence is yet to provide much tangible impact in drug target Mendelian randomization, it has offered some promise for functional annotation of genetic variants that may serve as instruments [84], to help inform on their mechanism of effect and suitability for any given study. Given the dramatic growth in our understanding of applications of artificial intelligence, it seems inevitable that more uses will emerge over the next few years. However, we believe that automation of drug target Mendelian randomization is still a while away. The nuanced interplay of biology, statistics, genetics, epidemiology, clinical translation, and drug development essential for successful implementation of the paradigm likely means that specialist and experienced skillsets will continue to be required, and such a combination is rarely amenable to automation.

## Conclusions

**Fig. 2:**
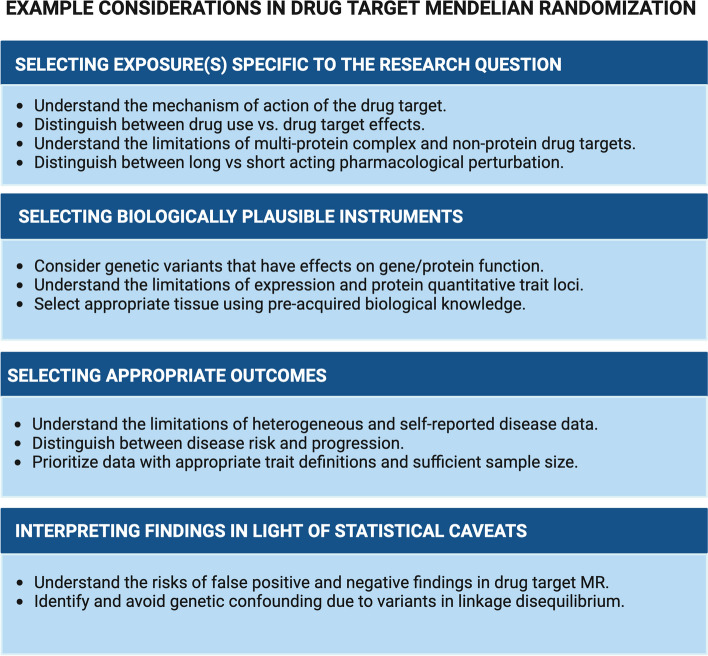
Some key considerations in drug target Mendelian randomization

We believe that it is through ongoing study of a greater diversity of pharmacological targets, therapeutic areas, and data sources that the field of drug target Mendelian randomization will continue to develop. Continual sharing of insights and learnings through scientific publications and support to methodologists and practitioners will enable the field to move forward efficiently (Fig. 2), bringing more successful drugs to market. Close collaboration with those that directly invest in and undertake drug development is also essential, to ensure that the findings can have maximum impact. All of this will serve individuals who stand to benefit from more effective medicines.

## Data Availability

No datasets were generated or analysed during the current study.

## Abbreviations

ACE: Angiotensin-converting enzyme
ANGPTL3: Angiopoietin-like protein 3
ANGPTL4: Angiopoietin-like protein 4
eQTL: Expression quantitative trait locus
GIPR: Glucose-dependent insulinotropic polypeptide receptor
LPL: Lipoprotein lipase
pQTL: Protein quantitative trait locus
